# Lethality in a Murine Model of Pulmonary Anthrax is Reduced by Combining Nuclear Transport Modifier with Antimicrobial Therapy

**DOI:** 10.1371/journal.pone.0030527

**Published:** 2012-01-26

**Authors:** Ruth Ann Veach, Jozef Zienkiewicz, Robert D. Collins, Jacek Hawiger

**Affiliations:** 1 Department of Microbiology and Immunology, Vanderbilt University School of Medicine, Vanderbilt University Medical Center, Nashville, Tennessee, United States of America; 2 Department of Pathology, Vanderbilt University School of Medicine, Vanderbilt University Medical Center, Nashville, Tennessee, United States of America; Institute Pasteur, France

## Abstract

**Background:**

In the last ten years, bioterrorism has become a serious threat and challenge to public health worldwide. Pulmonary anthrax caused by airborne *Bacillus anthracis* spores is a life- threatening disease often refractory to antimicrobial therapy. Inhaled spores germinate into vegetative forms that elaborate an anti-phagocytic capsule along with potent exotoxins which disrupt the signaling pathways governing the innate and adaptive immune responses and cause endothelial cell dysfunction leading to vascular injury in the lung, hypoxia, hemorrhage, and death.

**Methods/Principal Findings:**

Using a murine model of pulmonary anthrax disease, we showed that a nuclear transport modifier restored markers of the innate immune response in spore-infected animals. An 8-day protocol of single-dose ciprofloxacin had no significant effect on mortality (4% survival) of A/J mice lethally infected with *B. anthracis* Sterne. Strikingly, mice were much more likely to survive infection (52% survival) when treated with ciprofloxacin and a cell-penetrating peptide modifier of host nuclear transport, termed cSN50. In *B. anthracis*-infected animals treated with antibiotic alone, we detected a muted innate immune response manifested by cytokines, tumor necrosis factor alpha (TNFα), interleukin (IL)-6, and chemokine monocyte chemoattractant protein-1 (MCP-1), while the hypoxia biomarker, erythropoietin (EPO), was greatly elevated. In contrast, cSN50-treated mice receiving ciprofloxacin demonstrated a restored innate immune responsiveness and reduced EPO level. Consistent with this improvement of innate immunity response and suppression of hypoxia biomarker, surviving mice in the combination treatment group displayed minimal histopathologic signs of vascular injury and a marked reduction of anthrax bacilli in the lungs.

**Conclusions:**

We demonstrate, for the first time, that regulating nuclear transport with a cell-penetrating modifier provides a cytoprotective effect, which enables the host's immune system to reduce its susceptibility to lethal *B. anthracis* infection. Thus, by combining a nuclear transport modifier with antimicrobial therapy we offer a novel adjunctive measure to control florid pulmonary anthrax disease.

## Introduction

Pulmonary anthrax caused by inhaling *B. anthracis* spores represents a serious threat in biowarfare and bioterrorism. This threat is underscored by the accidental Sverdlovsk airborne outbreak in the former Soviet Union and more recent attempts to deliberately spread *B. anthracis* spores via the U.S. Postal Service in 2001 [1], [2]. Inhaled *B. anthracis* spores are disseminated throughout the body causing bacteremia, which is refractory to treatment with antibiotics and leads to extensive lung injury and death [3]. The cardinal features of lung injury involve hemorrhage in the mediastinum and pleural cavity, necrosis of mediastinal lymph nodes, and pulmonary edema with hyaline membrane formation. These life-threatening changes are due to the action of toxins secreted from vegetative forms of the bacilli. Two toxic effector proteins produced by bacilli, edema factor (EF) and lethal factor (LF), each form a binary complex with a pore-forming protective antigen (PA) [4].

The *B. anthracis* capsule and other conserved pathogen-associated molecular structures are recognized by Toll-Like Receptors (TLRs), the mainstays of innate immunity, initiating signaling pathways that determine the immune system response to bacterial infection [5]. Dendritic cells, which normally play a key role in this response to prevent the spread of anthrax bacteria from the site of infection throughout the body [6], are “disarmed” by LF and EF [7]–[9]. These toxic enzymes profoundly alter innate and adaptive immune responses that enable production of TNFα and other pro- and anti-inflammatory cytokines needed to fight infection [10]. Edema factor acts as a calcium- and calmodulin (CaM)-dependent adenylate cyclase that is 1,000 fold more active than mammalian CaM-activated adenylate cyclase [11]–[13]. It causes prominent edema at the site of infection, the inhibition of neutrophil function, and suppression of the production of TNFα and IL-6 by monocytes [14], [15]. EF-generated cAMP activates cAMP-dependent protein kinase A (PKA), which in turn phosphorylates cAMP response element binding protein (CREB) [14], [16] leading to suppression of mitogen-activated protein kinase (MAPK) kinase p38 [17] and inhibition of multiple transcription factors involved in cytokine production such as nuclear factor kappa B (NFκB) [18] and nuclear factor of activated T cells (NFAT) [19]. Lethal factor, a zinc metalloprotease, suppresses production of two effectors of innate immunity in macrophages, TNFα, and nitric oxide (NO), and reduces expression of other cytokine gene transcripts [20]–[22]. LF also inactivates MAPK kinase, leading to aberrant intracellular signaling [15] and contributing to the death of cultured macrophages [23]–[26]. Thus, anthrax toxins greatly affect the signaling to the nucleus essential for genome reprogramming in macrophages and dendritic cells.

Transcription factors are transported to the nucleus by the adaptors importins/karyopherins [10], [27]. These adaptors recognize the nuclear localization signal (NLS) on karyophilic proteins and thereby transport an array of signal transducers and transcription factors across the nuclear membrane [28]. Their “cargos” include, among others, NFκB, activator protein 1 (AP-1), CREB, and interferon regulatory factor 3 (IRF3) [29]. Previously, we demonstrated in a murine model of lethal shock induced with bacterial endotoxin that survival was increased from 0% to 90% by treatment with a cell-penetrating nuclear transport modifier [30]. Therefore, we postulated that modulating the nuclear shuttling of these and other transcriptional activators and repressors of innate and adaptive immunity might provide *in vivo* protection from overwhelming infection with *B. anthracis* spores.

To test the hypothesis that a nuclear transport modifier would display a protective effect in pulmonary anthrax, we selected a treatment protocol in which pulmonary anthrax was caused by a lethal dose of inhaled *B. anthracis* spores refractory to an 8-day treatment with the antibiotic ciprofloxacin. The cSN50 peptide employed in this model contains a cyclized form of the NLS from the p50/NFκB1 subunit of NFκB. The NLS was fused to the signal sequence-derived hydrophobic region from fibroblast growth factor 4. This hydrophobic segment serves as a membrane-translocating motif (MTM), which enables peptide or protein cargo to penetrate the plasma membrane of multiple cell types in various organs through a receptor/transporter- and endocytosis-independent mechanism [31]–[33]. NLS, as a cargo, competitively targets the adaptor Rch1/importin alpha 1/karyopherin alpha 2 [34], among other importins/karyopherins (Zienkiewicz J., Armitage A., and Hawiger J. unpublished), thereby modulating its nuclear shuttling function. We surmised that regulating nuclear transport of the overexpressed repressors of innate immunity generated by the high levels of cAMP induced by LF would help restore normal immune function. Concurrently, controlling nuclear transport of activators could prevent an excessive inflammatory response.

We now show that cell-penetrating nuclear transport modifier, cSN50, suppressed lung injury and lethality of pulmonary anthrax when mice exposed to 10 million spores of *B. anthracis* Sterne were also treated with the antibiotic ciprofloxacin. Adjunctive treatment with cSN50 restored components of the innate immune response and reduced levels of the hypoxia biomarker, erythropoietin (EPO). Lung edema and hemorrhagic lesions were ameliorated and survival was significantly increased. Thus, the addition of a nuclear transport modifier in antibiotic–treated pulmonary anthrax markedly reduced lethality in this model.

## Results

We tested our hypothesis that abnormal signaling to the nucleus is responsible for suppression of the innate immune response by anthrax toxins. Therefore, we examined the effect of a cell-penetrating peptide modifier of nuclear transport, cSN50, in an *in vivo* model of pulmonary anthrax treated with the antibiotic ciprofloxacin. To compensate for lower virulence of capsule –deficient *B. anthracis* Sterne, we employed an experimental model of pulmonary anthrax infection using A/J mice. These mice are C5 complement protein-deficient, thus being susceptible to *B. anthracis* infection with Sterne strain [35]. Consistent with prior studies [36], this experimental model of pulmonary anthrax via intranasal administration of *B. anthracis* spores was effective in delivering up to 70% of infectious units to the lungs.

Because our pilot experiments using monotherapy with cSN50 peptide alone to treat spore-infected mice did not improve survival but indicated a significant delay in time to death and a rise in serum levels of the chemokine monocyte chemoattractant protein-1 (MCP-1) (not shown), we designed a combination therapy by adding ciprofloxacin to treatment with the nuclear transport modifier. We chose a dosing schedule of ciprofloxacin that would partially control the rapid replication of anthrax bacilli in infected animals but not prevent the lethal outcome. Using a ciprofloxacin protocol that favored the lethal outcome provided a sufficient system for evaluating the impact of cSN50 peptide treatment on the course of infection while controlling rapid replication of bacilli with antibiotic.

We evaluated the consequences of adjunctive treatment with a nuclear transport modifier not only by survival but also by monitoring the dynamic changes in mediators of innate immunity (cytokines and chemokine) and the hypoxia biomarker EPO. These mechanistic studies were complemented by histologic examination of the extent of lung injury and involvement of other organs. This analysis also allowed us to monitor the spread or containment of *B. anthracis* toxin-producing vegetative forms. Results of the experiments presented below establish the hitherto unrecognized role of nuclear transport modifier in correcting deranged innate immune and hypoxia responses in this model of inhalational anthrax, thereby significantly increasing survival of anthrax spore-infected animals.

### Increased Survival of Mice Challenged with *B. anthracis* Spores and Treated with Nuclear Transport Modifier cSN50 and Ciprofloxacin

**Figure 1:**
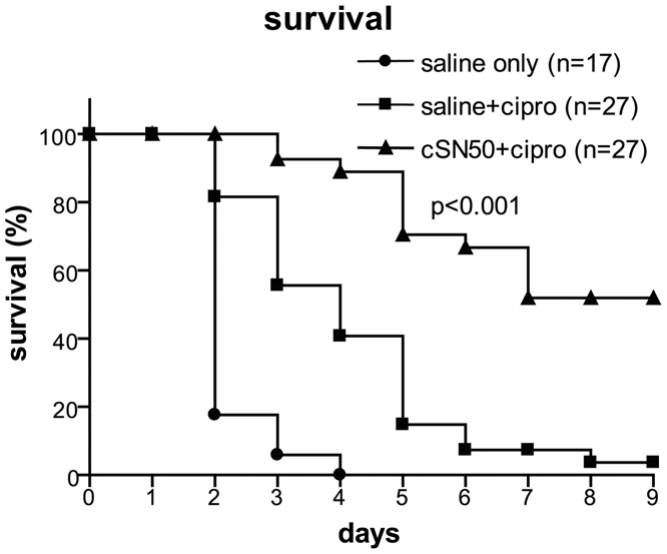
Survival in inhalational anthrax was increased by combination of cSN50 with ciprofloxacin. Female A/J mice were infected intranasally (IN) with 10^7^ *B. anthracis* spores and treated with 15 injections of cSN50 during the first 2 days and daily ciprofloxacin (triangles) or saline and ciprofloxacin (squares) or saline without ciprofloxacin (circles). The *p* value represents the significance of the difference in survival between the two ciprofloxacin-treated groups (with and without cSN50 peptide).

W tested a cell-penetrating nuclear transport modifier, cSN50 peptide, for its effect on survival of *B. anthracis* spore-challenged mice treated with ciprofloxacin. Each mouse was infected with 10^7^ spores and treated with multiple intraperitoneal (IP) injections of either cSN50 or saline. Twenty four hours after exposure to spores, daily administration of ciprofloxacin was begun and continued for 8 days. We titrated the doses of spores and ciprofloxacin so that death was delayed but not prevented. Untreated control mice were given IP saline injections, but no ciprofloxacin. These mice died between 2 to 4 days post-infection (Fig. 1), while 50–70% of the infectious spore dose was recovered from the lungs of representative animals sacrificed one hour post-infection. Prior to death, mice developed labored respirations, most likely due to lung and pleural injury noted at autopsy. Mice receiving ciprofloxacin and saline lived longer, but ultimately all but one succumbed to infection within 9 days (4% survival). In contrast, a significant number of mice receiving a combination of nuclear transport modifier, cSN50, and ciprofloxacin survived (Fig. 1). Survival at the ninth day in three independent experiments was 52% (p<0.001). At that time point, survivors were euthanized to evaluate the extent of lung injury. Four mice treated with cSN50 and ciprofloxacin were observed for 21 days without signs of recurrent *B. anthracis* infection. Thus, nuclear transport modifier cSN50 enhanced the survival following suboptimal antibiotic treatment of mice exposed to *B. anthracis* spores.

### *In Vivo* Levels of Cytokines, Chemokine, and Erythropoietin in *B. anthracis*- Infected Mice

**Figure 2:**
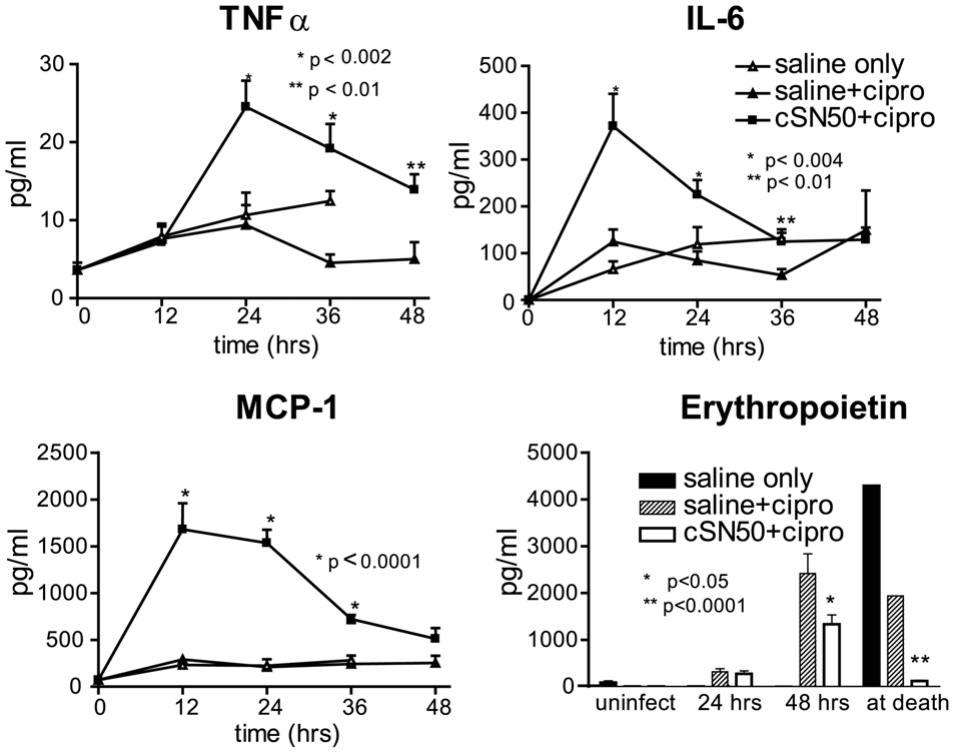
cSN50 restored depressed cytokine/chemokine responses to *B. anthracis* infection and attenuated a rise in erythropoietin, a hypoxia biomarker. Blood serum levels of TNFα, IL-6, and MCP-1 were measured in female A/J mice infected with 10^7^ *B. anthracis* spores IN and treated with cSN50 and daily ciprofloxacin (solid squares) or saline and ciprofloxacin (solid triangles) or saline without ciprofloxacin (open triangles) as in Fig. 1. Blood serum levels of Erythropoietin were measured in the same mice: cSN50 and ciprofloxacin (white), saline and ciprofloxacin (diagonal lines) or saline without ciprofloxacin (black). Error bars indicate the+S.E. of the mean value in mice represented by each data point. The *p* values represent the significance of the differences between the two ciprofloxacin-treated groups.

Innate immune response to non-toxin-producing mutants of *B. anthracis* is manifested by the production of proinflammatory cytokines. In contrast, lung dendritic cells infected with toxin-producing strains of *B. anthracis* display a striking paucity of cytokine production reflecting suppression of the innate immune response [6]. Consistent with these prior observations, mice receiving saline only or saline and ciprofloxacin following *B. anthracis* spore challenge showed depressed levels of TNFα, IL-6, and MCP-1. Surprisingly, treatment with cSN50 and ciprofloxacin restored induction of these mediators of innate immunity in *B. anthracis*-infected animals (Fig. 2). Other pro- and anti-inflammatory cytokines, IL-12p70, interferon gamma (IFNγ), and IL-10 remained unchanged (not shown). Increased hypoxia, manifested by an elevated level of its biomarker, erythropoietin (EPO), has been previously reported in mice challenged with anthrax lethal toxin [37]. Consistent with these prior findings, EPO was elevated at 48 h after *B. anthracis* spore infection and at death in control mice that received saline only or saline and ciprofloxacin. In contrast, this hypoxia marker was attenuated in all mice receiving treatment with cSN50 and ciprofloxacin (Fig. 2). Thus, nuclear transport modifier suppressed hypoxia associated with the lethal outcome of pulmonary anthrax.

### Histologic Analysis of Head, Lungs and Other Organs in *B. anthracis*-Infected Mice

**Figure 3:**
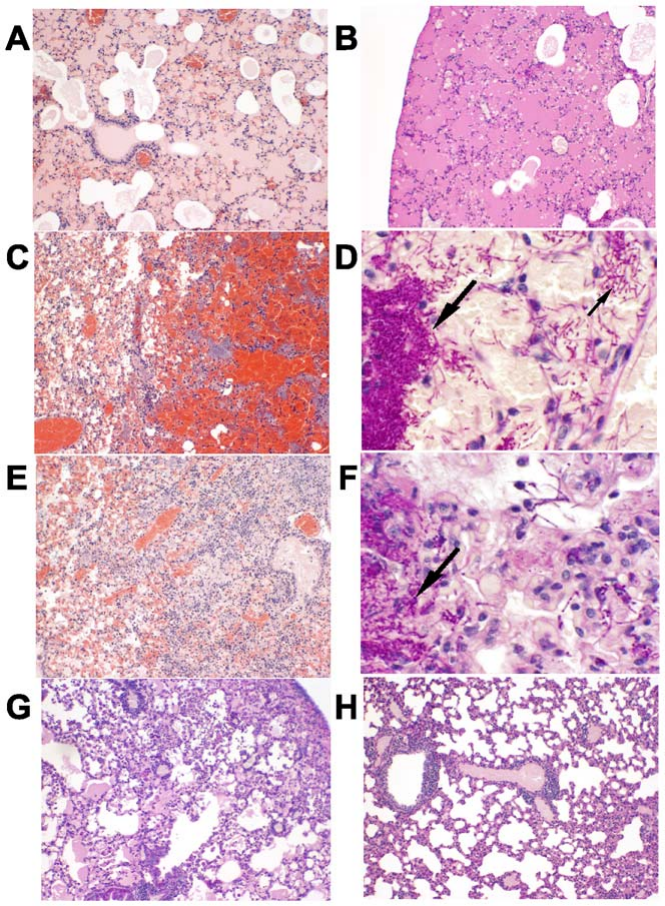
Lung injury in mice challenged intranasally with *B. anthracis* was reduced by cSN50 treatment. Lung sections A, C, E, G and H stained with Hematoxylin and Eosin (HE). B, D, and F stained with Periodic Acid-Schiff and Hematoxylin (PAS). A–D. Untreated mice. Marked pulmonary edema (A–B) and hemorrhage (C) throughout. Clumps of bacteria (large arrow) and individual bacteria (small arrow) highlighted by PAS stain (D). E–F. Mice treated with saline+ciprofloxacin. Foci of edema, cellular infiltrates, and hemorrhage (E). Clumps of bacteria (arrow) and scattered individual bacteria visualized with PAS stain (F). G–H. Mice treated with cSN50 peptide+ciprofloxacin. Minimal edema in mice surviving 9 days (G) and essentially normal lungs in mice sacrificed at 21 days (H). PAS stained sections in mice from these groups were negative for bacilli (not shown).

There were no signs of infection in the sinuses or brain of untreated control mice infected IN with *B. anthracis,* and very few spores were detected in the sinus cavities of mice sacrificed one hour after instillation, indicating that in this model infection is primarily initiated by spores reaching the upper airways. Untreated mice showed marked pulmonary edema (Fig. 3A–B), hemorrhage (Fig. 3C) and clumps of *B. anthracis* bacilli were prominent in lung tissue (Fig. 3D). There were also many *B. anthracis* bacilli in the vessels of the heart, kidneys, spleen, and liver indicating bacteremic spread of *B. anthracis* vegetative forms (not shown) as observed in the victims of the bioterrorism-related inhalational anthrax outbreak and in other animal studies. The PAS staining in Fig. 3B showed the extent of edema. In the lungs of mice receiving ciprofloxacin alone, foci of edema and hemorrhage were found (Fig. 3E), along with clumps of *B. anthracis* vegetative forms as shown with PAS stain in Fig. 3F. In contrast, surviving mice treated with cSN50 peptide and ciprofloxacin showed minimal pulmonary edema at the ninth day of observation (Fig. 3G) and lungs were essentially normal in mice euthanized at 21 days (Fig. 3H). PAS-stained sections in mice from these groups of survivors were negative for *B. anthracis* vegetative forms in all organs examined, and very few spores were detected in lung sections stained with a modified Ziehl's carbol fuchsin dye for spores in tissues (not shown). In mice that succumbed to infection, there were fewer stained spores seen in lungs of cSN50-treated animals compared to those treated with ciprofloxacin alone. Thus, the cytoprotective effect of cSN50 peptide in the lungs correlated with the survival of mice challenged with 10^7^ *B. anthracis* spores and receiving ciprofloxacin also. Moreover, the beneficial effect of cSN50 peptide added to ciprofloxacin facilitated lung clearance of *B. anthracis* bacilli and prevented their spread to other organs (the heart, spleen, liver, and kidneys).

## Discussion

These results show that a nuclear transport modifier restored the markers of the innate immune response and prevented the florid toxicity of pulmonary anthrax. While the once-daily dosing schedule of ciprofloxacin used here can lead to development of antibiotic-resistant organisms in as little as one day after infection [38], this only accentuates the ability of cSN50 to potentiate the innate immune system to limit the progression and lethal outcome of pulmonary anthrax. However, ciprofloxacin, through its ability to reduce anthrax biofitness by inducing error-prone replication at the suboptimal dosage used in this model, may have acted in synergy with cSN50 and thereby enhanced its apparent effect. Whether this interaction is needed for the cSN50 effect and whether nuclear transport modulation would be equally effective in conjunction with alternative antibiotic treatments has not yet been determined. Among the first 10 cases of bioterrorism-related inhalational anthrax reported in the United States in 2001 survival was 60% [39]. Those who succumbed were presenting fulminant signs of illness when they were first treated with antibiotics directed toward *B. anthracis*. Whether adjunctive therapy that restores innate immunity and affords cytoprotection can attenuate fulminant signs of illness in ultimately fatal cases remains to be investigated.

Cell-penetrating nuclear transport modifier has a broad anti-inflammatory and cytoprotective spectrum. *In vivo* cytoprotection by cSN50 peptide has been previously demonstrated in endotoxic lipopolysaccharide (LPS) and staphylococcal enterotoxin B (SEB) models. These models are characterized by overproduction of proinflammatory cytokines and chemokines in acute lung inflammation as well as acute liver inflammation associated with massive apoptosis and hemorrhagic necrosis [40]–[42]. Moreover, Shiga toxin 2-induced endothelial cell injury was attenuated with nuclear transport modifier SN50 [43]. Subsequently, we found that in mice treated with cSN50 peptide, expression of hundreds of LPS-inducible and LPS-suppressed genes were modulated in the liver and spleen (Li C., Liu XY., Sims L., Levy S., and Hawiger, J. unpublished results). Thus, cSN50 peptide has a bimodal effect on regulation of genes that encode mediators of innate immune function depending on the cell type and the nature of the microbial pathogen. In pulmonary anthrax, the unlocking effect of cSN50 peptide on suppressed innate immunity mediators (TNFα, IL6, and MCP-1) is consistent with bimodal regulation of genome-wide response by this nuclear transport modifier.

The complex interplay of *B. anthracis* virulence factors incapacitates macrophages, dendritic cells, T cells, and natural killer T cells [7], [44], [45]. Therefore our findings with cSN50 indicate its restorative action on innate immune responses in anthrax toxin-deranged cells by unlocking expression of TNFα, IL6, and MCP-1. These mediators of innate immunity are regulated by CBP/p300; therefore the exceedingly high levels of cyclic AMP generated by EF [11]–[13] can dysregulate the activities of the CREB family of basic leucine zipper transcription factors that act as both transcriptional activators and repressors depending on the cell type and stimulus [46]. A member of the CREB/ATF family, activating transcription factor 3 (ATF3) is a negative regulator of genes that encode proinflammatory cytokines IL-6 and IL-12 [47]. ATF3 was transcriptionally upregulated in lethal toxin-challenged cultured mouse macrophages [48]. Nuclear translocation of this and other repressors of cytokine genes is required for their genome regulatory activity [29]. Our previous studies have established the role of cSN50 in regulating nuclear transport of stress-responsive transcription factors NFκB, NFAT, AP-1, and STAT1 [10], [34] while unpublished results indicate that cSN50 is able to affect nuclear trafficking of ATF3, hypoxia-induced factor 1 (HIF-1), IRF3 and CREB as well (Veach RA. and Hawiger J. unpublished results).

In summary, we have demonstrated that a cell-penetrating modifier of nuclear transport, cSN50 peptide, offers a promising adjunctive therapy to the treatment of pulmonary anthrax. Given its rapid delivery and therapeutic potential, this cell-penetrating nuclear transport modifier may represent a more favorable strategy for a combination treatment of systemic anthrax than previously reported vaccine and monoclonal antibodies.

## Materials and Methods

### Ethics Statement

All animal handling and experimental procedures were performed in accordance with the American Association of Accreditation of Laboratory Animal Care guidelines and approved by the Institutional Animal Care and Use Committee of Vanderbilt University. The laboratory animal care program of VU (PHS Assurance #A3227-01) has been accredited by AAALAC International since 1967 (File #000020). Mice are closely monitored to minimize the amount of pain experienced by the animals, and those that exhibit end-stage symptoms are euthanized as soon as it is apparent they will not recover.

### Peptide Synthesis and Purification

MTM-containing peptide cSN50 was synthesized, purified, filter-sterilized, and analyzed as described previously [30], [34].

### *Bacillus anthracis* culture and spore preparation

*Bacillus anthracis* Sterne strain was grown in 2×SG medium (nutrient broth supplemented with 2 mM Magnesium Sulfate, 27 mM Potassium Chloride, 1 mM Calcium Nitrate, 100 µM Manganese Chloride, and 700 nM Ferrous Sulfate) at 37°C with constant shaking (300 rpm) until sporulation (5–7 days). The culture was centrifuged for 7 min at 8000 g at 4°C, resuspended in sterile water, heated at 65°C for 1 h to kill vegetative bacilli and germinated spores then washed 3 times with sterile water [49]. Phase microscopy confirmed that the resulting preparation contained >95% refractile spores. The number of colony forming units (cfu) was determined by plating serial dilutions of spores on Luria broth (LB) agar and counting the *B. anthracis* colonies. Spores were aliquoted in 20% glycerol and stored at −80°C. The number of cfu was reconfirmed before each use.

### Pulmonary Anthrax Model in Animals

Female A/J mice (7–8 weeks) were purchased from the Jackson Laboratories (Bar Harbor, ME), and randomly assigned to study groups. For *B. anthracis* Sterne spore instillation, mice were anesthetized by intraperitoneal (IP) injection of 50 mg/kg Nembutal, and 1×10^7^ spores in 50 µl saline were administered to each mouse intranasally (IN). The number of spores reaching the lungs was determined in each experiment. Briefly, 2–3 mice were sacrificed 1 h post-infection and lungs were removed under sterile conditions, homogenized, and serial dilutions of homogenates plated on LB agar. By this method we determined that 50–70% of the instilled dose of spores reached the lungs of infected mice. Mice that received doses of 10^5^ or 10^6^ spores IN were observed for 9 days and all were asymptomatic and survived (not shown). In contrast, all mice that received 10^7^ spores died within 4 days with signs of overt infection (see Results and Discussion).

### Animal Treatment Protocol

Female A/J mice were injected IP with 200 µl saline or cSN50 peptide (3.5 mg/ml) at 30 minutes before and 30 minutes, 1.5 h, 2.5 h, 3.5 h, 6 h, 9 h, 12 h, 15 h, 18 h, 21 h, 24 h, 30 h, 36 h, and 42 h after IN infection with 10^7^ spores of *B. anthracis* Sterne strain. Treatment with ciprofloxacin, 50 mg/kg, was administered subcutaneously (s.c.), beginning 24 h after spore challenge and continuing once daily for 8 days. Blood was collected from the saphenous vein before infection, at 12, 24, 36 and 48 h after spore challenge, and at death. Serum was separated from clotted blood and stored at −20°C. All animals were observed for morbidity and mortality for up to 9 days and some cSN50 peptide-treated mice were monitored for up to 21 days after spore challenge. Moribund animals were humanely euthanized by IP injection of pentobarbital. Surviving animals were euthanized in the same manner 9 or 21 days after spore challenge. Organs and heads of mice were collected at death and immersed in 10% formalin for histologic analysis (see below).

### Cytokines, Chemokine, and Erythropoietin Assays in Serum

We measured IL-6, TNFα, IFNγ, IL-10, IL-12, and MCP-1 in serum by a Cytometric Bead Array according to the manufacturer's instructions (BD Biosciences). Murine erythropoietin (EPO) was measured by enzyme-linked immunosorbent assay (ELISA) according to the manufacturer's instructions (R and D Systems). Results are expressed as the mean+S.E.

### Histological Analyses

Organ samples (lungs, liver, spleen, heart, and kidney) and heads (skin removed) were collected at death from mice who succumbed to *B. anthracis* infection and from surviving mice euthanized after 9 or 21 days. Formalin-fixed, paraffin-embedded sections were stained with hematoxylin and eosin (HE) or periodic acid-Schiff and hematoxylin (PAS) to assess damage from infection and presence of *B. anthracis* vegetative forms, and with a modified Ziehl-Neelson dye, using hot carbol fuchsin at 60 degrees for 4 minutes followed with a methylene blue counterstain, to detect spores in tissues [50], [51].

### Statistical Analyses

A log rank test was used for statistical analysis of survival. A one-way analysis of variance, a two-way repeated measure analysis of variance and the Student's *t* test were used to determine the significance of the difference in the levels of cytokines, chemokine, and erythropoietin.
